# Research trends in transient receptor potential vanilloid in cardiovascular disease: Bibliometric analysis and visualization

**DOI:** 10.3389/fcvm.2023.1071198

**Published:** 2023-02-22

**Authors:** Lingfeng Zhang, Yantao Xu, Yingxu Ma, Tianjian Xie, Chan Liu, Qiming Liu

**Affiliations:** ^1^Department of Cardiovascular Medicine, The Second Xiangya Hospital of Central South University, Changsha, China; ^2^Xiangya School of Medicine, Central South University, Changsha, China; ^3^Department of Dermatology, Xiangya Hospital, Central South University, Changsha, China; ^4^International Medical Department, The Second Xiangya Hospital, Central South University, Changsha, China

**Keywords:** transient receptor potential vanilloid, TRPV, cardiovascular disease, CVD, bibliometric analysis methods

## Abstract

**Background:**

Transient receptor potential vanilloid (TRPV) is one of the transient receptor potential protein groups; cardiovascular system disease is a crucial cause of mortality among people globally.

**Objective:**

This article is intended to accomplish a bibliometric analysis of the trends and public interest since TRPV was reported for the first time.

**Methods:**

The article summarized the Web of Science (WOS) Core Collection on the relationship between TRPV and cardiovascular system disease each year from 2000 to 2021. Data extraction and visualization were completed by R package bibliometrix. Keyword citation burst and co-citation networks were generated and produced by CiteSpace. The map evaluating the distribution of country and region was painted in GunnMap 2 (lert.co.nz). The ranking was performed using the Standard Competition Ranking method. Co-authorship and co-occurrence were analyzed with VOSviewer.

**Results:**

After removing duplicated data, books, conference proceedings, and articles of uncertain age, 493 were included, and 17 were excluded. The pattern of publication years showed that the number of publications increased rapidly from 2008 to 2021 with no peak in the number of publications until 2021. The geographical distribution pattern revealed a considerable gap in the number of publications between the United States, China, and other countries, with East Asian institutions leading the world in this area. The pattern of co-authorship showed that 77 institutions were divided into 19 clusters, each covering one country or region.

These results suggest that intercontinental cooperation among institutions should be strengthened. The core authors section displayed the change in the most published authors. Keyword analysis listed six burst keywords. Co-citation analysis of references from 2011 to 2021 showed the number and centrality of citations to leading articles.

**Conclusion:**

Our findings reveal trends and public interest in transient receptor potential vanilloid for cardiovascular disease. These findings suggest that the field has experienced significant growth since 2008, with the United States and China in dominant positions. Our findings also suggest that intercontinental cooperation should be strengthened, and that future research hotspots may focus on pharmacological mechanisms and in-depth exploration of drug clinical trials and new clinical disease application areas such as hypertension, diabetes, and cardiac arrhythmias, which could serve as a foundation for further research.

## Introduction

Cardiovascular disease (CVD) has been a global threat to allage groups, mainly middle-aged and elderly individuals, for several decades. In 2019, an estimated 17.9 million CVD deaths occurred worldwide (1). The number of fatalities has also continued to rise over the past 30 years, reaching 12.3 million in 1990. Fortunately, most cardiovascular diseases can be attributed to behavioral risk factors such as tobacco and alcohol abuse, unhealthy diet and obesity, and physical inactivity, and it is estimated that up to 90% of these diseases can be prevented (2). Transient receptor potential vanilloid (TRPV) is a members of the transient receptor potential protein group that regulates calcium ions and senses heat and inflammation by activating vanilloid receptors. TRPV was first discovered in *Caenorhabditis elegans* in 1997 (3). As research has progressed, it has been found that TRPV plays a role in cardiovascular diseases, such as cardiac failure, arrhythmogenesis, and pulmonary arterial hypertension (4).

## Methods

### Data sources and search strategy

Bibliographic data were obtained from the Web of Science Core Collection (WOScc). The search strategy designed is that: (TS = (Cardiovascular) OR TI = (Cardiovascular) OR AB = (Cardiovascular)) AND (TS = (TRP channels) OR TI = (TRP channels)) OR (AB = (TRP channels) OR TS = (transient receptor potential channels) OR TI = (transient receptor potential channels) OR AB = (transient receptor potential channels)) OR TS = (transient receptor potential vanilloid) OR TI = (transient receptor potential vanilloid) OR AB = (transient receptor potential vanilloid) OR (TS = (TRPV) OR TI = (TRPV) OR AB = (TRPV)) (TS = Topic; TI = Title; AB = Abstract). Limitations were English, original research, and review, and all the documents were filtered between January 01, 2000 and December 31, 2021.

**Figure 1:**
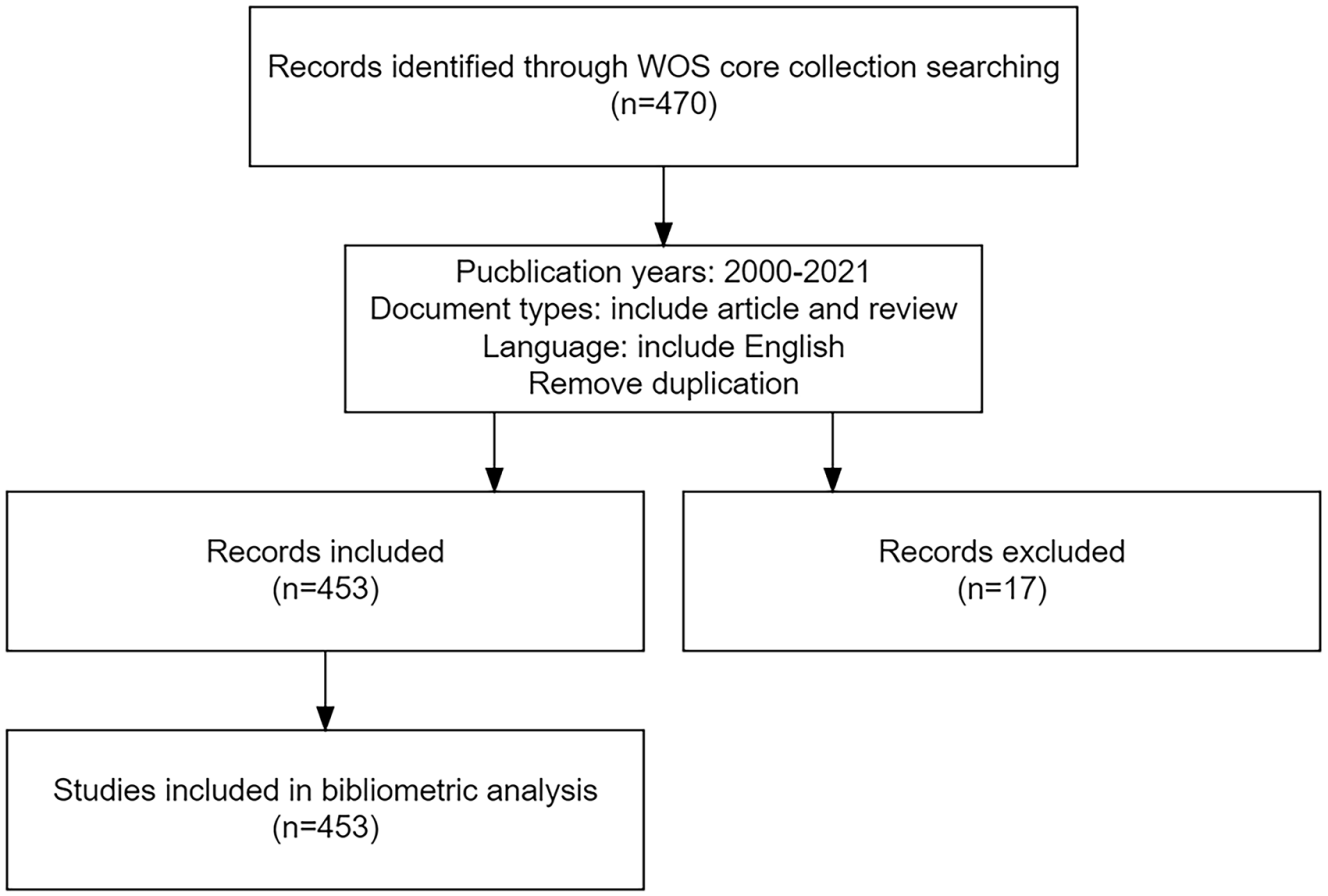
Flowchart showing the steps to identify and filter papers. Publication years were limited from 2000 to 2021. The language was limited to English.

Four hundred seventy articles were extracted from the Web of Science Core Collection. Book chapters, meeting abstracts, proceeding papers, editorial material, and early access were eliminated, leaving 453 documents for the bibliometric analysis and visualization. A flowchart presented more details. The search was completed on August 14th, 2022 (Figure 1).

### Data extraction and analysis

The following characteristics were included in the retrieval publication year, country and region, origin (including organization and institution), publication source, core authors, keywords, and primary references. A detailed search strategy was provided in Multimedia Appendix 1. Bibliometric analysis and visualization were performed using VOSviewer (version 1.6.18, Leiden University), bibliometrix package in R (version 4.2.1, R Foundation), and CiteSpace. SCImago Graphica (version Beta 1.0.23) was used to create a graph illustrating the number of posts in different regions. GunnMap 2 (lert.co.nz) was used to assess geographical differences in distribution. The ranking was determined using the standard competition ranking method. Co-authorship analysis, co-occurrence analysis, and visualization were conducted using VOSviewer.

## Results

### Distribution of publications

**Figure 2:**
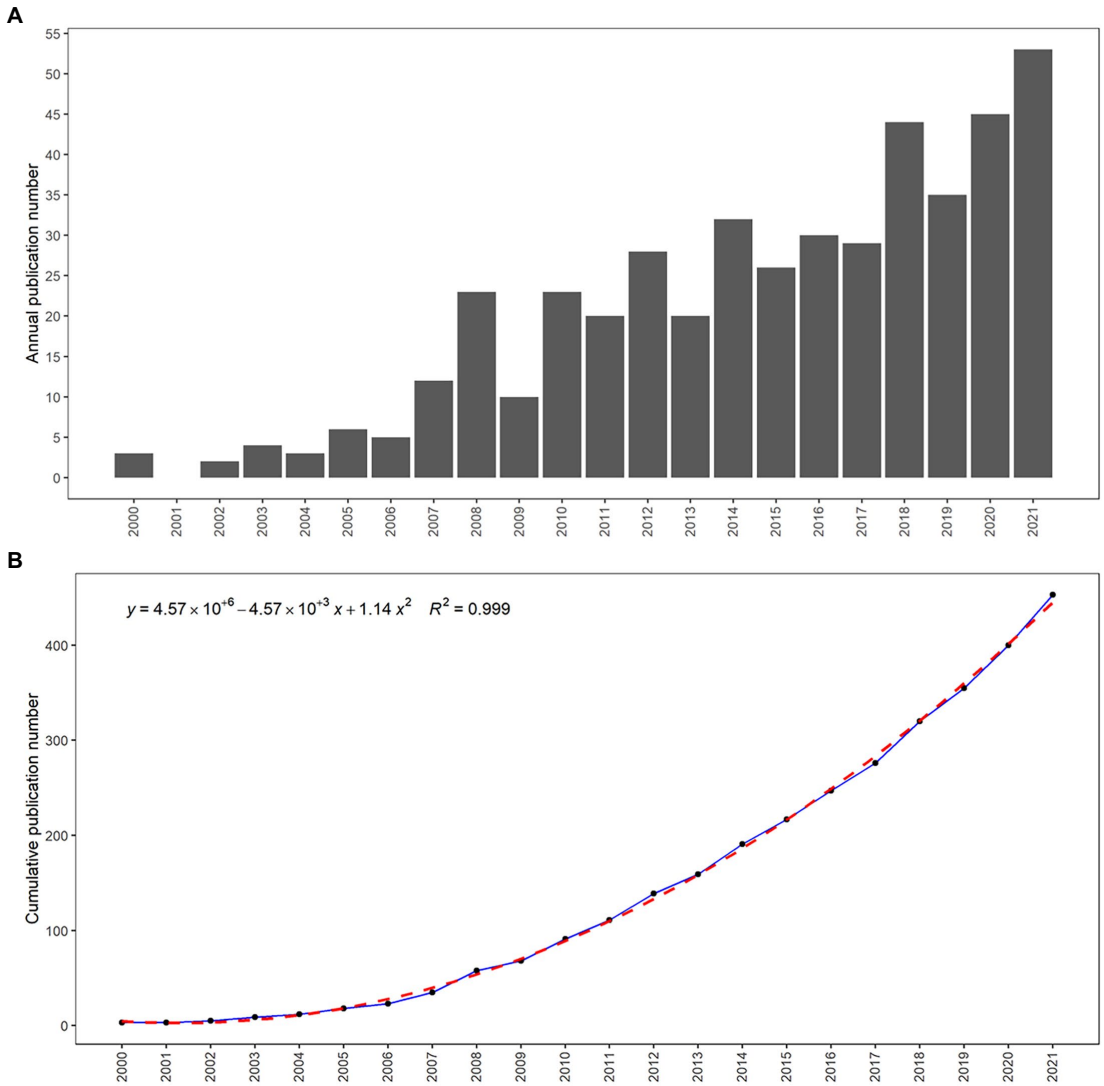
Distribution of chronological publication **(A)**. The number of publications accumulated and the number per year **(B)**.

The bar chart showed the chronological distribution (Figure 2A). From 2000 to 2006, the number of publications per year increased steadily, with a near-exponential growth trend, but did not reach a peak yet. A dramatic increase followed this in the number of publications per year from 2007 to 2008 and 2009 to 2010. Figure 2B graphically showed the total number of cumulative publications. Chronologically, the growth of publications on the specific topic shows a relatively slow growth rate in the cumulative number of publications from 2000 to 2006, and a sharp increase from 2006 to 2008. A peak briefly occurred in 2008 and then did not occur until 2021.Overall, the number of publications and their growth rate continued to grow steadily from 2000 to 2021. The annual percentage growth rate of publications is 14.7.

**Figure 3:**
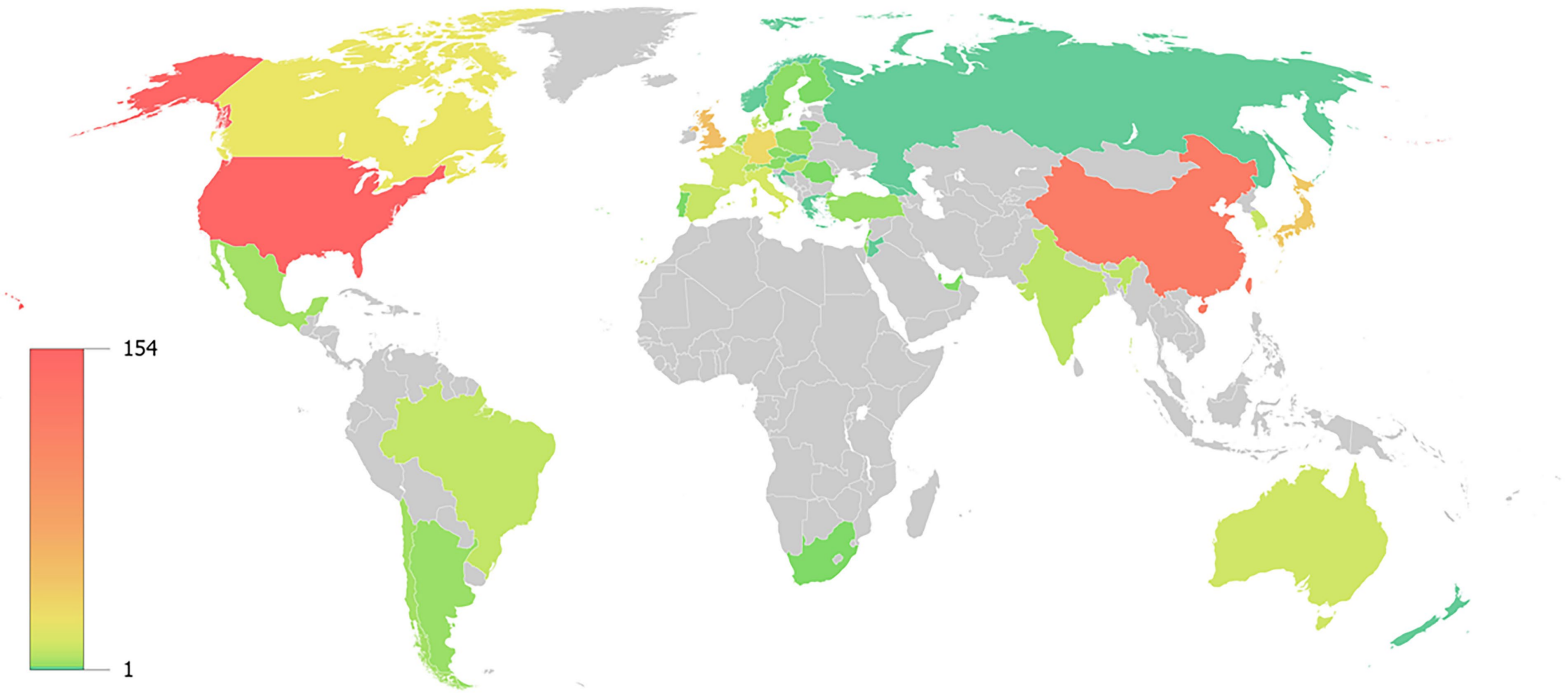
Geographical distribution of 50 countries is depicted, with the color bar on the left being linear.

**Figure 4:**
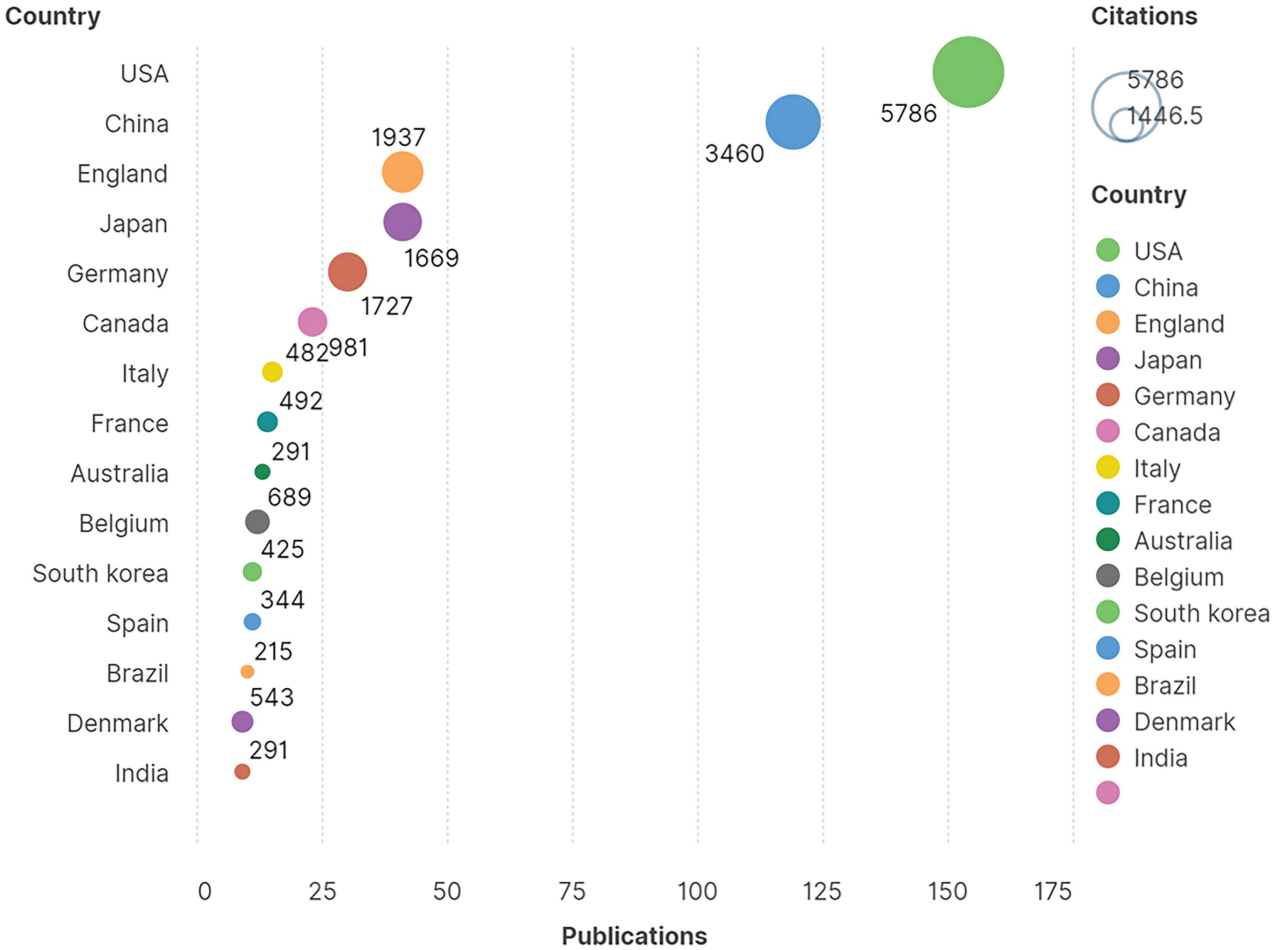
The 15 most prolific countries and regions were listed with the number of publications and citations.

According to the geographical distribution pattern, 453 articles were published from 50 countries and regions. A heatmap depicted the 50 countries and regions that published articles with green representing a value to 1, and red representing a value close to 154 (Figure 3). The 10 countries with the most publications were listed in Table 1. Overall, the United States had the highest number of publications at 154 out of 453, or 34.00%, far surpassing China with 119 out of 453 publications, or 26.27%. England and Japan both had 41 out of 453 publications, or 9.05%. In terms of citations, the United States and China were far ahead. Interestingly, the United States and China had the highest number of publications and also had a larger percentage of citations than the sum of the 3rd to 15th countries, comprising 60.26% of all publications and 41.19% of all citations, respectively (Figure 4).

**Table 1:** The 10 most productive organizations.

	Organization	Country	Articles	Citations	Total link strength^a^
1	Third Military Medical University	China	14	593	46
2	Fourth Military Medical University	China	10	480	47
3	Kyushu University	Japan	10	348	30
4	Chinese University of Hong Kong	China	9	717	40
5	China Medical University	China	7	84	9
6	Kyoto University	Japan	7	277	6
7	Pennsylvania State University	United States	7	214	4
8	University of Leeds	United Kingdom	7	343	19
9	Fukuoka University	Japan		474	36
10	National Institute of Environmental Health Sciences	United States		390	10

a The total link strength in VOSviewer represents all links between a given node and other nodes, and it indicates how the entry interacts with other entries. A non-negative number indicates the strength of a link. If a node is not linked to other nodes, then the total strength of the link is equal to zero.

### Analysis of leading organizations and public sources

**Figure 5:**
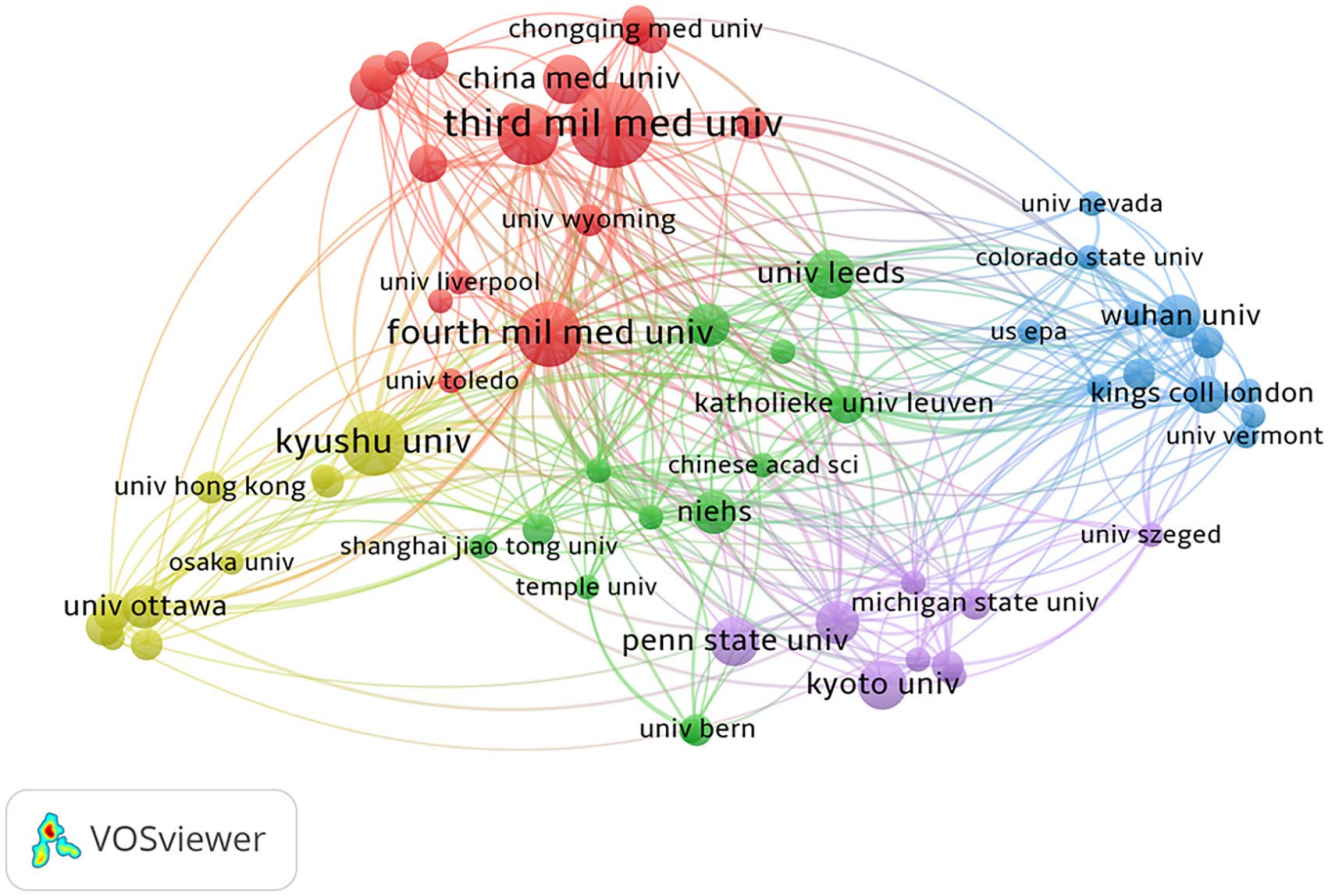
Co-authorship analysis of organizations. Co-authorship analysis of organizations showed by plots. It was normalized in the fractionalization method and weighted by the number of publications. The thickness of the lines indicates the strength of co-authorship relationships. Different clusters were painted in different colors.

The publishing organization information was analyzed with VOSviewer. There were 453 articles contributed by 660 institutions. After merging duplicate organizations and excluding irrelevant ones, 21 organizations that met the inclusion threshold were visualized. The 10 organizations with the most published articles were listed in Table 1. The 10 organizations with the most published articles are listed in Table 1. The most influential organization was the Third Military Medical University with 14 out of 453 publications, or 3.09%, followed by the Fourth Military Medical University with 10 out of 453 publications, or 2.21%, and Kyushu University with 10 out of 453 publications, or 2.21%. Among the most prolific organizations, 4 out of 10 were from China, and 3 out of 10 were from Japan. In total, 7 out of 10 were from East Asian organizations, which was far more than the 2 from the United States that published the most documents. A co-authorship analysis of the organizations was also conducted (Figure 5). It revealed that all 21 most published institutions were grouped into five clusters, each roughly representing the core organization from an East Asian country. The size of the nodes in the graph indicates the frequency of occurrence. We selected some of the most frequent institutions, which indicates that these institutions have a strong presence in the cluster and are representative. The red, yellow, green, blue, and purple clusters included Third Military Medical University and Forth Military Medical University in China, Kyushu University in Japan, Katholieke University from Taiwan, China, Wuhan University in China, and Kyoto University in Japan, in, respectively. The National Institute of Environmental Health Sciences (NIEHS) and the University of Leeds in the green cluster were two of the few influential institutions from western countries. This result showed that it was evident that East Asian organizations were the dominant leader in this topic and that intercontinental cooperation among various institutions should be strengthened, especially for institutions in the United States, England, and Germany, as the United States had the most significant number of publications but had a mismatched number of core institutions.

After analyzing published materials, the 10 most published journals, along with their impact factor (IF) in 2020 and 2021 for research on the role of TRPV in the cardiovascular disease were extracted (Table 2). The British Journal of Pharmacology (impact factor 9.473), with 19 documents, was the most prolific journal, ranking first. The American Journal of Physiology-Heart and Circulatory Physiology (18 publications, IF 5.125) and the International Journal of Molecular Sciences (13 publications, IF 6.208) followed as the second and third most published journals, respectively. The impact factor of the 10 journals ranged from 3.493 for Channels to 13.081 for Cardiovascular Research. These findings suggest that Cardiovascular Research may be the most influential journal in in the field of TRPV and cardiovascular disease.

**Table 2:** The 10 most published journals.

Rank	Journal	Publications	Impact factor (2020)	Impact factor (2021)
1	British Journal of Pharmacology	19	8.740	9.473
2	American Journal of Physiology-Heart and Circulatory Physiology	18	4.733	5.125
3	International Journal of Molecular Sciences	13	5.924	6.208
4	Cardiovascular Research	12	10.787	13.081
5	European Journal of Pharmacology	12	4.432	5.195
6	Frontiers in Physiology	12	4.566	4.755
7	Hypertension	10	10.190	9.897
8	Channels	8	2.581	3.493
9	Cell Calcium	7	6.817	4.690
10	Journal of Biological Chemistry	7	5.157	5.486

### Analysis of co-authorship and core authors

The data of co-authors was analyzed using VOSviewer. 2,510 authors contributed to a total of 453 publications.

**Figure 6:**
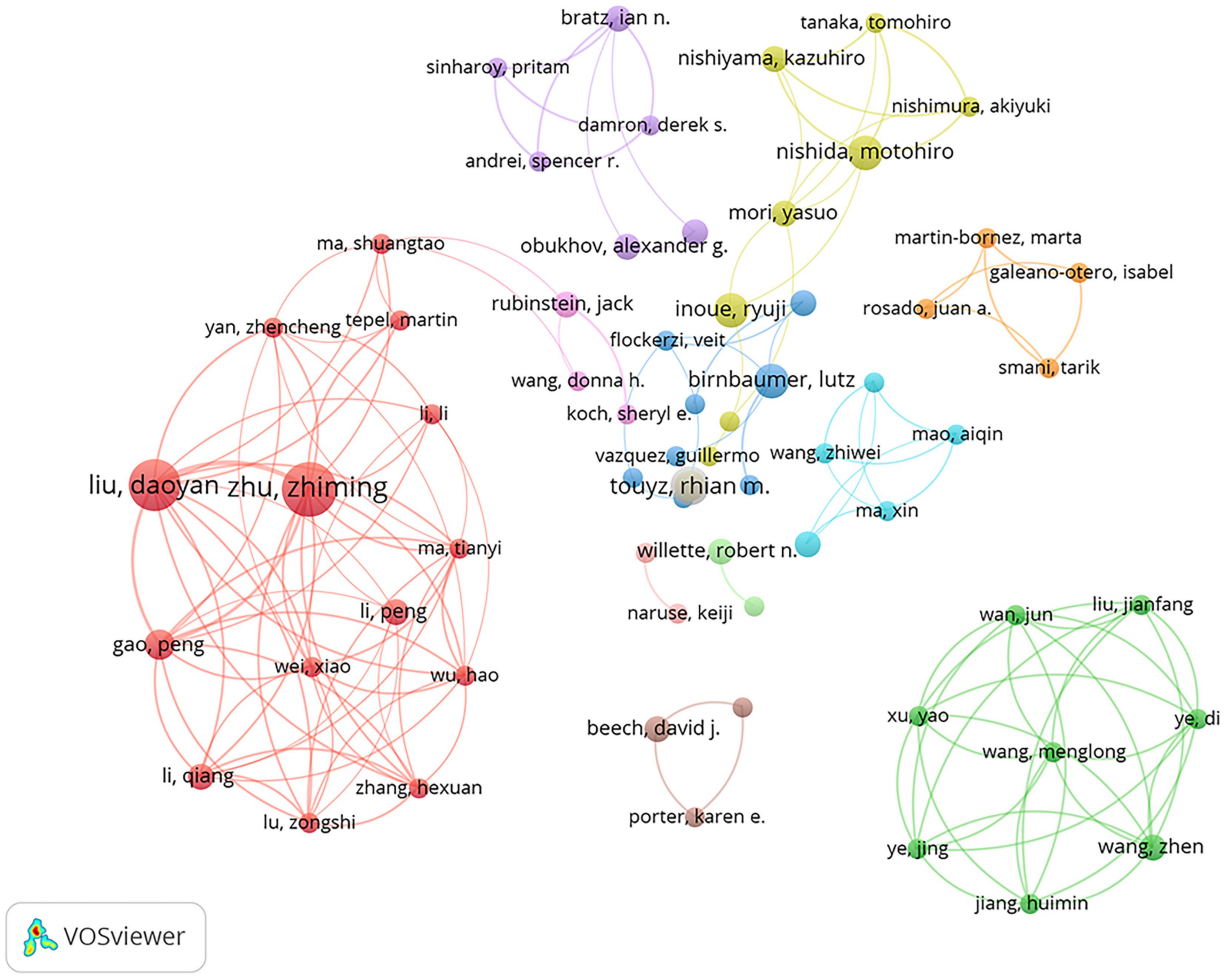
The co-authorship analysis depicts the 77 institutions grouped into 19 clusters.

The most important evaluation criteria for core authors included the number of publications, total citations, and H-index. Based on these criteria, the most productive authors were identified and visualized (as shown in Table 3; Figure 6). Zhu Zhiming, director and professor of Cardiology and Endocrinology at Third Military Medical University and Hypertension and Endocrinology at the Center for Hypertension and Metabolic Diseases at Daping Hospital, Army Medical University, was the most productive author in this field, having published 11 articles and received a total of 564 citations. Liu Daoyang, professor of the department of Hypertension and Endocrinology at the Center for Hypertension and Metabolic Diseases at Daping Hospital, Army Medical University, was the second most productive author with 10 publications and 504 total citations. Rhian M. Touyz is the Executive Director and Chief Scientific Officer of the Research Institute of the McGill University Health Centre. Three of the 10 most cited authors were from Hypertension and Endocrinology at the Center for Hypertension and Metabolic Diseases at Daping Hospital, Army Medical University (formerly known as Third Military Medical University before 2017): Zhu Zhiming, Liu Daoyan, and Gao Peng. It is clear that Hypertension and Endocrinology at the Center for Hypertension and Metabolic Diseases at Daping Hospital, Army Medical University (formerly known as Third Military Medical University before 2017) was a dominant player in this research field.

**Table 3:** Top 10 core authors with the most published articles.

Rank	Author	Organization	Documents	Citations	H Index
1	Zhu, Zhiming	Third Military Medical University (China)	11	564	7
2	Liu, Daoyan	Third Military Medical University (China)	10	504	6
3	Touyz, Rhian M.	University of Glasgow (United Kingdom)	7	427	3
4	Birnbaumer, Lutz	National Institute of Environmental Health Sciences (United States)	6	390	5
5	Inoue, Ryuji	Fukuoka University (Japan)	6	474	4
6	Nishida, Motohiro	Kyushu University (Japan)	6	310	1
7	Earley, Scott	University Nevada (United States)	5	242	4
8	Gao, Peng	Third Military Medical University (China)	5	125	5
9	Moccia, Francesco	University of Pavia (Italy)	5	68	5
10	Beech, David J.	University of Leeds (United Kingdom)	5	295	/

**Figure 7:**
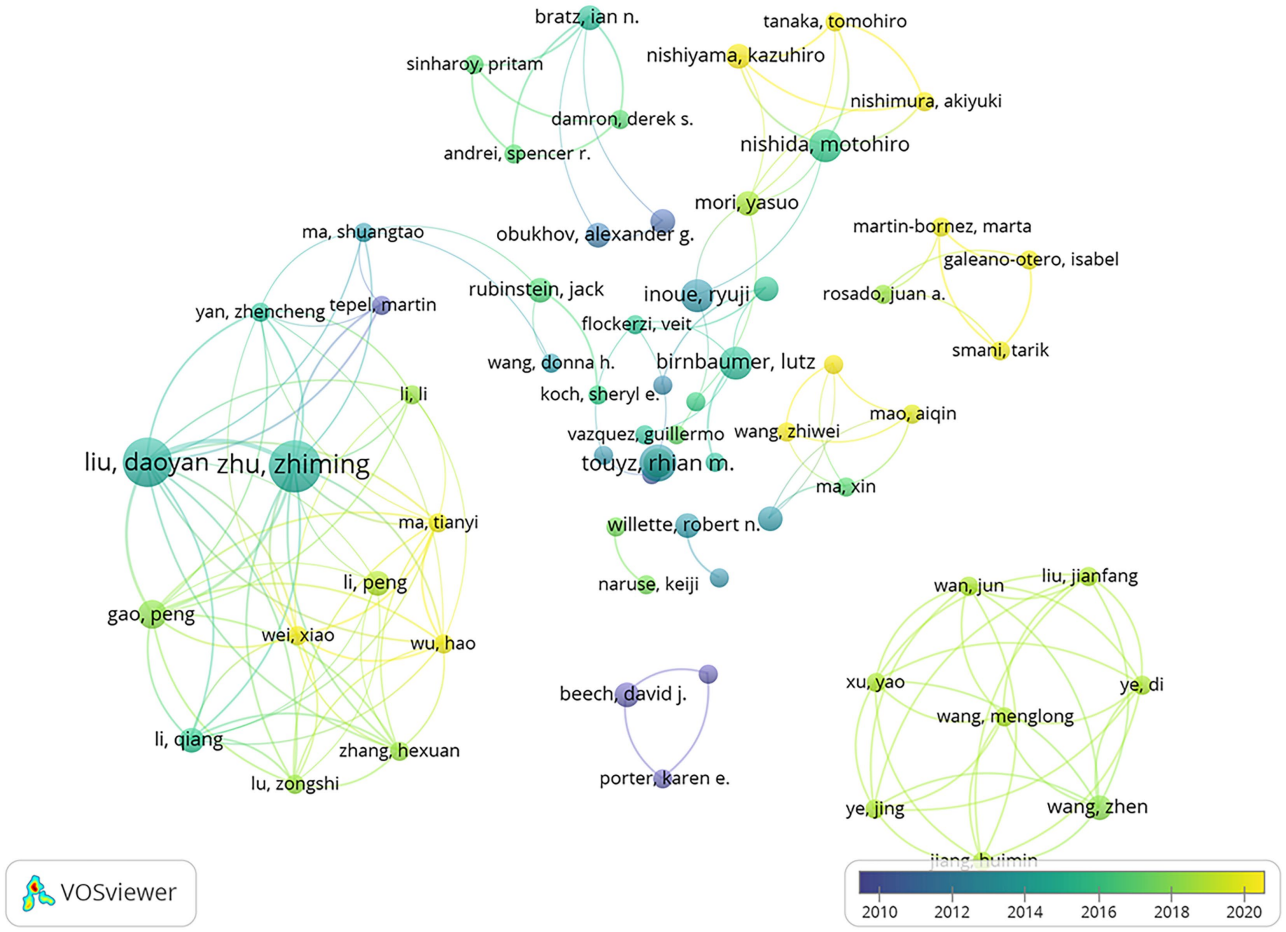
Overlay visualization of co-authorship analyzed in the method of Association strength, weighted by citations, scored by average year of publication. The strength of the relationships is indicated by the thickness of the lines. The average year of publication is represented by the color of the circle.

The results showed that the top 10 most prolific authors came from East Asia, Europe, and the United States. It was unusual for East Asian researchers, particularly those from Third Military Medical University, to have the same level of influence as researchers from Europe and the United States. It is possible that the smaller number of data may have skewed the results. Figure 7 is an overlay visualization of the co-authorship relationship among 2,510 authors, 270 of which met the inclusion threshold. The figure showed that the most influential authors, such as Zhu, Zhiming, and Liu, Daoyan had close collaborations. The average year of publication, which represents the average of all relevant publications by these authors, indicates the time period in which the authors were most active on the topic. The figure showed that the average year of publication ranged from 2016 to 2018. Some other authors, such as Moccia Francesco, published more actively after 2020 and were likely to become leaders in the future.

### Analysis of keywords and burst terms

**Figure 8:**
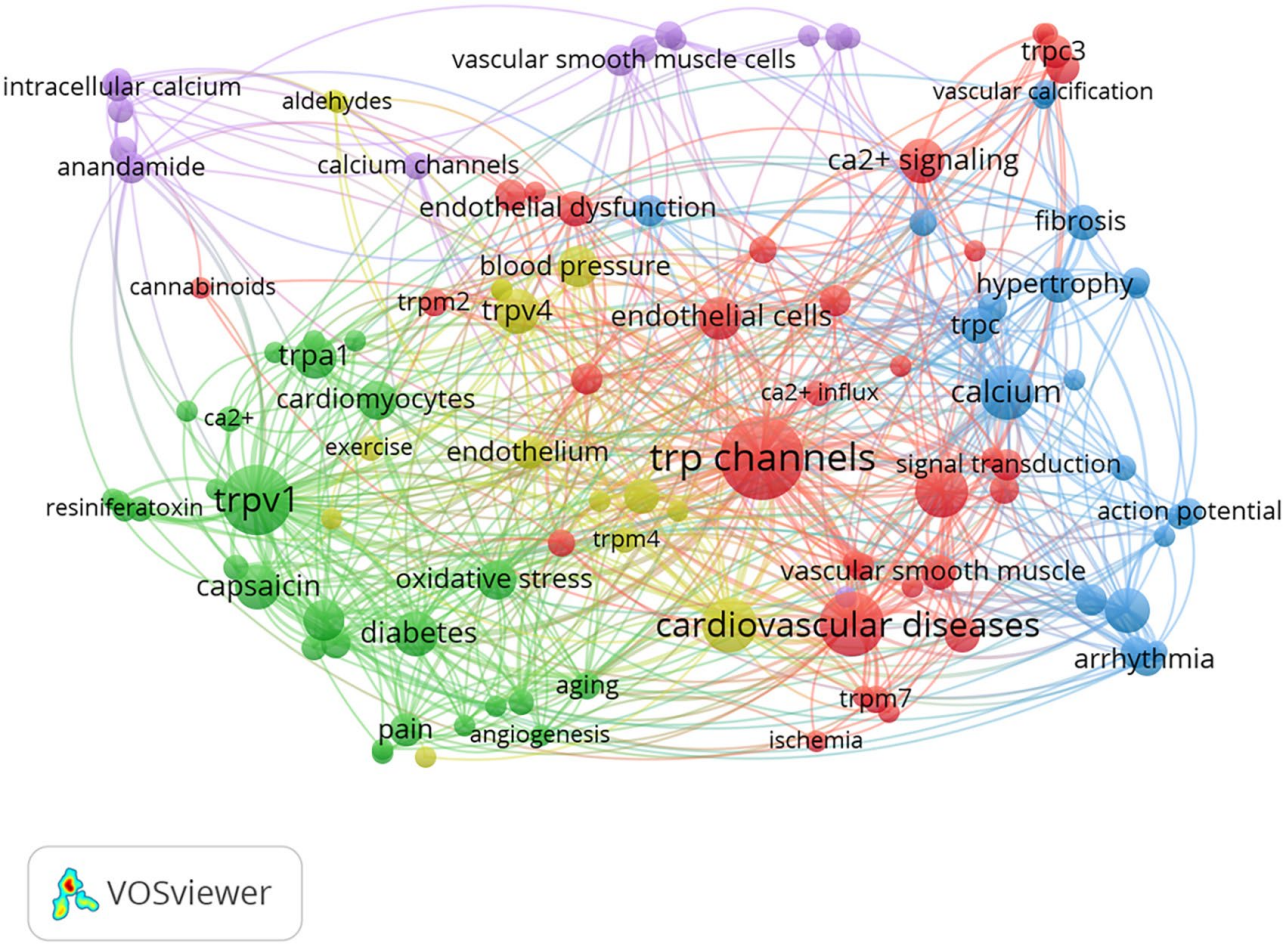
The co-occurrence analysis of keywords was shown by the co-occurrence of keywords normalized in the Linlog/modularity method, weighted by an occurrence for each plot.

**Figure 9:**
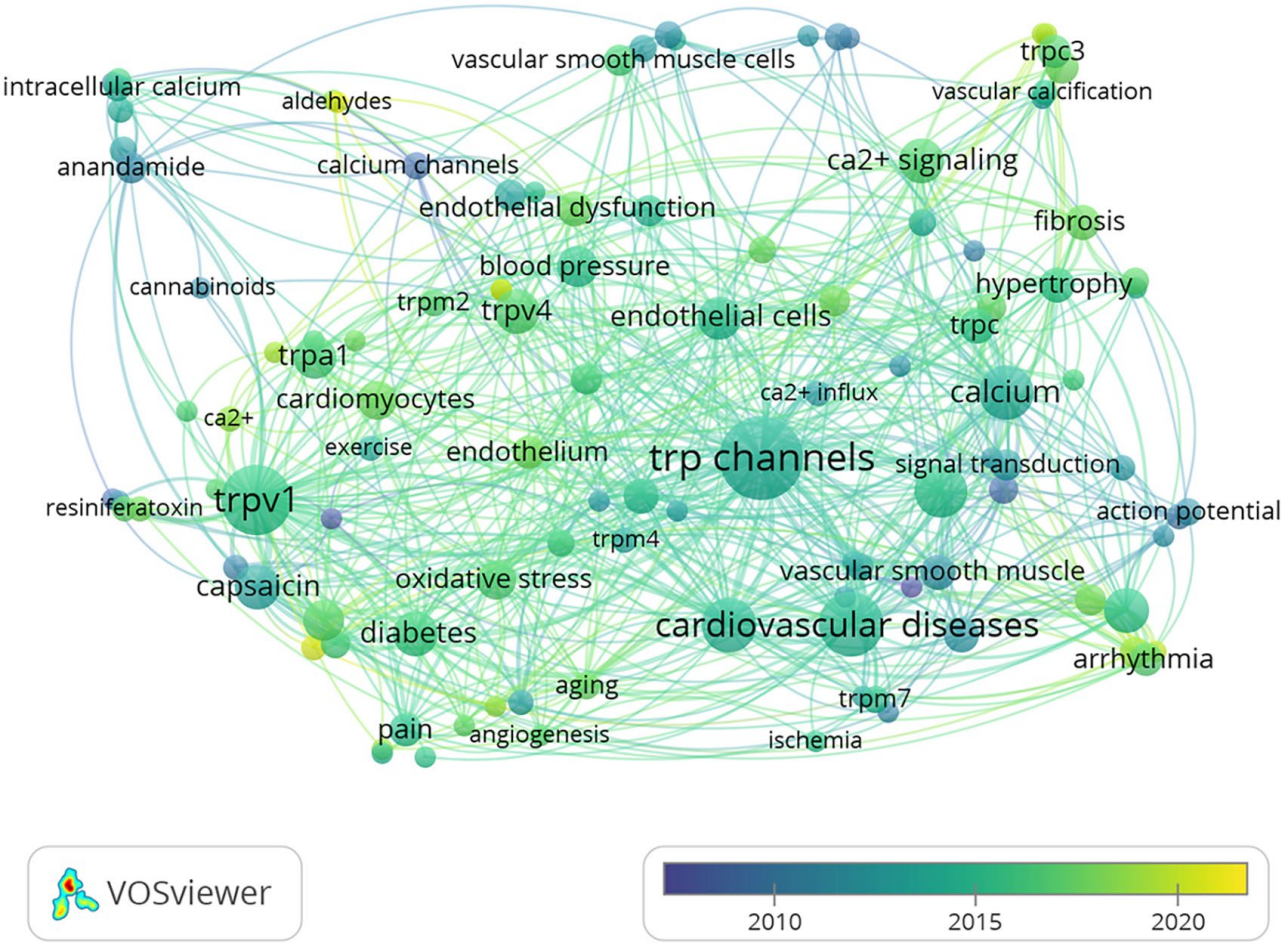
These keywords were grouped by year of publication, and the color of the circles represented the average year.

All 453 documents were analyzed to extract 1,339 keywords. The co-occurrence of these keywords was analyzed using overlay visualization and a network of author-given keywords after duplicates were removed. Of the 1,339 keywords, the 113 most frequently occurring ones that met the inclusion threshold were divided into 2 clusters (shown in Figure 8) and grouped by publication date (between 2012 and 2018; shown in Figure 9). The range of publication dates corresponds to the high-growth phase of publication seen in Figure 2. The keywords were distinguished into five main clusters for the network. The green cluster included keywords related to transient receptor potential channels activated by vanilloid chemicals and metabolism dysfunction in cells or the body, such as “TRPV1,” “capsaicin,” “obesity,” “diabetes,” “apoptosis,” “oxidative stress,” “TRPA1,” “cardiomyocytes,” and “metabolic syndrome.” This may suggest that TRPV1 affects metabolism through apoptosis. The yellow cluster included terms related to vascular dysfunction and inflammation, such as “hypertension,” “blood pressure,” “endothelium,” “TRPV4,” and “inflammation,” which may indicate that TRPV4 is associated with vascular dysfunction and inflammation. The blue cluster included terms related to vascular and heart remodeling and related disease and TRPC, such as “calcium,” “heart failure,” “arrhythmia,” “hypertrophy,” “TRPC,” “vascular remodeling,” “atherosclerosis.” This may suggest that these vascular and heart remodeling processes have more links with TRPC. The purple cluster included terms r cannabinoid substances and proliferation, such as “anandamide,” “calcium channels,” “proliferation,” “endocannabinoid,” and “vascular muscle cells.” The red cluster included terms related to TRP channels and factors influencing cardiovascular disease in cells or the body, such as “TRP channels,” “ion channel,” “cardiovascular disease,” “endothelial cells,” “endothelial dysfunction,” “hydrogen peroxide,” “nitric oxide.”

**Figure 10:**
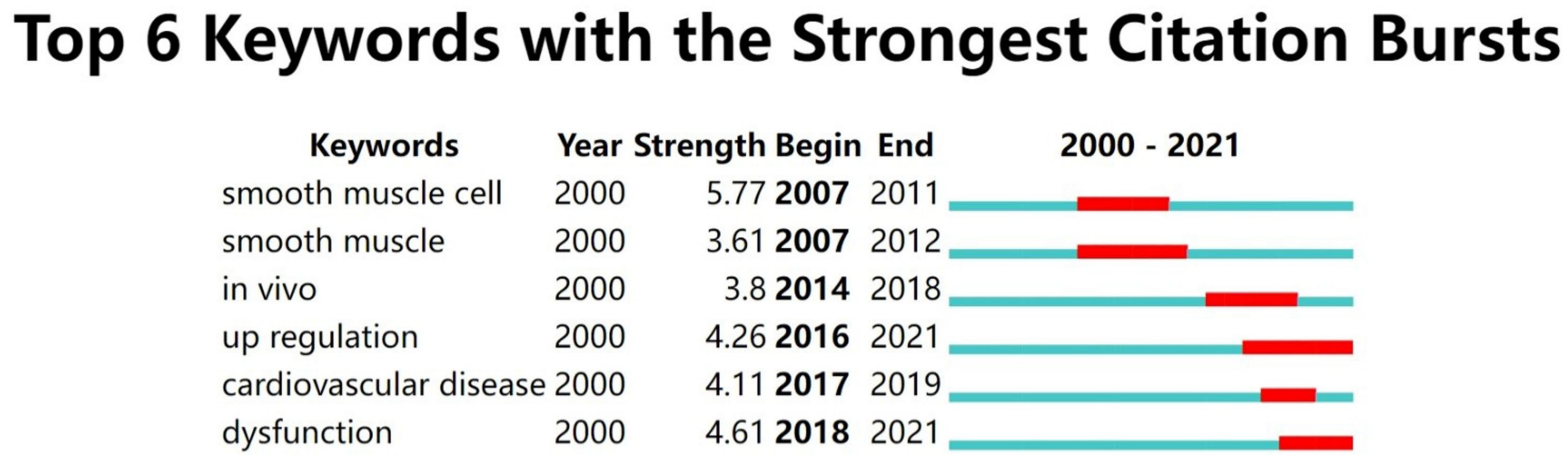
The 6 most vital frequency bursts keywords. Many frequency bursts indicate that a variable has changed significantly in a short period. The red bars indicate the duration of the bursts.

Burst keywords analyzed with CiteSpace. Figure 10 showed six keywords with the highest frequency burst of change, indicating a significant keyword change in a short period. In these six words, “smooth muscle cell” and “dysfunction” were either histologic or pathologic level keywords with the most muscular strength. For hot years, “smooth muscle cell” continued from 2007 to 2012 and “dysfunction” continued from 2018 to 2021, suggesting possible pathological mechanisms. The order of “smooth muscle cell,” “smooth muscle,” “*in vivo*,” and “cardiovascular disease” might reveal the research process from the cellular to the system level. “Up regulation” and “dysfunction” were sustained research hot spots for the near 5 years; both were future research hotspots in this field.

### Citation analysis

The citation counts of publications were mainly extracted using bibliometrix package. The 10 most cited publications were listed in Table 4. In total, the number of citations ranged from 198 to 314. The article published in the Pharmacological Reviews: “Transient receptor potential channels as drug targets: from the science of basic research to the art of medicine,” ranked first and had 314 total citations. Only two of the 10 most cited articles were original articles (“H2S and NO cooperatively regulate vascular tone by activating a neuroendocrine HNO-TRPA1-CGRP signaling pathway,” published in *Nature Communications* and “Activation of TRPV1 By Dietary Capsaicin Improve Endothelium-Dependent” published in *Cell Metabolism*). These two high-quality articles were published in sub-publications of Cell or Nature, demonstrating an extraordinary research level. The rest eight articles were all reviews. Undoubtedly, reviews were cited more often because of their comprehensive feature. Five of the eight reviews were published in journals of the pharmacologic category, meaning that TRPV research is mainly in the field of pharmacology. Although “H2S and NO cooperatively regulate vascular tone by activating a neuroendocrine HNO-TRPA1-CGRP signaling pathway” was the latest publication of 10, it had forth ranked citations, which also proves that it is a high-quality paper.

**Table 4:** The 10 most cited publications.

Rank	Title	DOI^a^	Source	Publication date	Total citations^b^
1	Transient receptor potential channels as drug targets: from the science of basic research to the art of medicine (13)	10.1124/pr.113.008268	Pharmacological Reviews	Jul 2014	314
2	Transient Receptor Potential Channels in Cardiovascular Function and Disease (14)	10.1161/01.RES.0000233356.10630.8a	Circulation Research	Jul 2006	297
3	Recent advances in the study of capsaicinoids and capsinoids (15)	10.1016/j.ejphar.2010.09.074	European Journal of Pharmacology	Jan 2011	285
d4	H2S and NO cooperatively regulate vascular tone by activating a neuroendocrine HNO-TRPA1-CGRP signaling pathway (16)	10.1038/ncomms5381	Nature Communications	Oct 2019	268
5	Physiology and pathophysiology of canonical transient receptor potential channels (17)	10.1096/fj.08-119495	Faseb Journal	Jul 2014	252
6	Transient receptor potential (TRP) channels: a clinical perspective (18)	10.1111/bph.12414	British Journal of Pharmacology	May 2014	227
7	Activation of TRPV1 by dietary capsaicin improves endothelium-dependent vasorelaxation and prevents hypertension (19)	10.1016/j.cmet.2010.05.015	Cell Metabolism	Apr 2017	224
8	Recent developments in vascular endothelial cell transient receptor potential channels (20)	10.1161/01.RES.0000187473.85419.3e	Circulation Research	Oct 2005	207
9	Systemic activation of the transient receptor potential vanilloid subtype 4 channel causes endothelial failure and circulatory collapse: Part 2 (21)	10.1124/jpet.107.134551	Journal of Pharmacology and Experimental Therapeutics	Aug 2008	200
10	Unraveling the mystery of capsaicin: a tool to understand and treat pain (22)	10.1124/pr.112.006163	Pharmacological Reviews	Oct 2012	198

a DOI, Digital Object Identifier.

b Total citations were until the end of December 2021.

**Figure 11:**
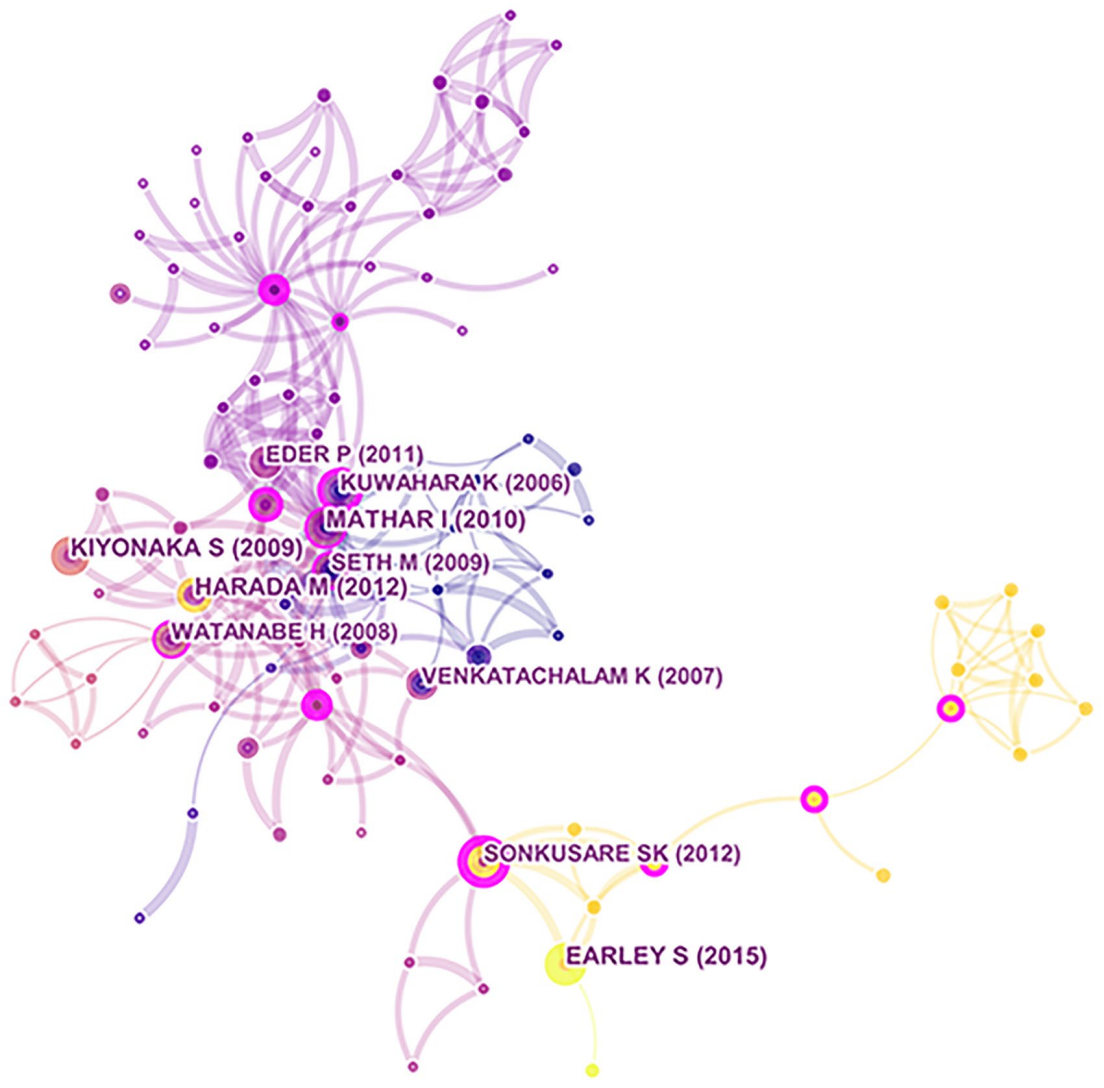
Co-citation analysis of references. Using VOSviewer, depict a co-citation analysis of references from 2010 to 2020. In VOSviewer, the size of a circle indicates number of citations. The purple area of the circle indicates the centrality of a document.

Co-citation references from 2011 to 2021 were analyzed and visualized by CiteSpace (version 5.8R3; Figure 11). In Figure 11, the size of a circle represents the number of citations, and the purple area of the circle depicts the centrality. The analysis showed that there was no dominant centrality but a few inferior centralities, such as Eder (5), Kuwahara (6), Mathar (7), Harada (8), Watanabe (9), Venkatachalam (10), Sonkusare (11), and Earley (12). The co-citations were largely dispersed.

## Discussion

### Principal findings

This study presents the most recent systematic information about the role of TRPV in cardiovascular disease. It provides an overview of this research area and identifies potential future areas of focus for interested researchers. The study included a comprehensive search of the Web of Science Core Collection for published literature on this topic that was published before December 31, 2021. A total of 453 bibliographies were retrieved and analyzed using bibliometric techniques. The analysis of chronology showed that from 2000 to 2006, the annual number of publications increased slowly, with no clear research trends. However, there was a sharp increase in the annual number of publications from 2007 to 2008 and 2009 to 2010, likely due to the discovery and study of several members of the TRPV channel family between 1998 and 2008 (23–28). As the study of TRPV channels continues to grow, especially following the recognition of this topic with a Nobel Prize, it is likely that we will see even more research in this field in the future.

The United States is the leading country in terms of the number of publications on TRPV and cardiovascular disease, but most of this literature is produced in collaboration with institutions in East Asia. China is the second most productive country, with East Asian countries leading the field overall. Other countries and regions, such as the United Kingdom, Japan, and European countries, have also made significant contributions. Despite the high number of publications from the United States, East Asian institutions and authors are more productive in this field. Of the 10 most productive organizations, seven are from East Asian countries (four from China and three from Japan), and five of the 10 most productive authors are from East Asia (three from China and two from Japan). The Third Military Medical University in China is particularly dominant, with three of the 10 most cited authors affiliated with the institution. Cardiovascular Research is the most influential journal on this topic in terms of the number of publications and impact factor (IF). In 2021, it published two TRPV-related papers, one of which was about how omega-3 fatty acids improve flow-induced vasodilation by enhancing TRPV4 in arteries from diet-induced obese mice. The other was an editorial about the first paper. The IF of the top 10 core journals has generally increased, with the exception of Channels. However, it is difficult to conclude that the impact of all journals has increased due to the overall increase in IF of journals in recent years due to the COVID-19 pandemic.

The results of the network analysis showed that most collaborations among authors occurred within the same continent, particularly within the same institution or in East Asia. There was a low level of collaboration between continents. These findings indicate that there is a need to improve intercontinental cooperation. In addition, the analysis of keywords and burst keywords revealed significant changes in research focus. There was a shift from studying action potentials, calcium channels, and anandamide to focusing on cardiomyocytes, endothelium, arrhythmias, diabetes, and oxidative stress. This suggests that research has moved from examining basic molecular mechanisms to studying the physiological and pathological mechanisms of specific diseases, and from focusing on smooth muscle and smooth muscle cells to examining upregulation and dysfunction. This indicates that theories are becoming more advanced and that there is increasing interest in exploring the role of TRPV in cardiovascular disease. The discovery of the TRPV channel family, including TRPV1, in 1998 marked the beginning of research in this field. However, it wasn’t until the subtypes and mechanisms of these channels were fully understood in the years following 2008 that research on TRPV channels began to accelerate. Currently, the focus of research has shifted from understanding the mechanisms of action of these channels to exploring their pharmacological mechanisms, with small molecule antagonists of channels like TRPV1, TRPV3, and TRPA1 entering clinical trials (18). As the study of TRPV channels continues to grow, especially following the recognition of this topic with a Nobel Prize, it is likely that we will see even more research in this field in the future.

### Strengths and limitations

This analysis provided more comprehensive and intuitive information than a literature review due to its use of quantitative statistical analysis and visualization. But it also has some limitations: (1) some minor subtopics have not been addressed or reflected due to the breadth of the TRPV field; (2) newly published articles are not cited in a timely manner; (3) our analysis focused on English manuscripts and data from non-English sources were ignored; (4) in addition to the quality of the articles, the number of citations can be influenced by a number of factors, including post-merger deletions, the tendency not to cite competitors or conflicting results, attitudes toward citing high IF core journals and attitudes toward citing review articles rather than original research, and country or language preferences; (5) this analysis included fewer studies, which may prone to statistical error and bias; and (6) only the citations and abstracts of the literature were analyzed and may have missed some essential information in the main text, such as the authors’ perspectives, outlook on the field, and their forward-looking opinions on the field.

## Conclusion

This paper focuses on the role of TRPV in the cardiovascular system and analyzes the most recent articles in the field of TRPV over the last two decades, including their publication years, regional distribution, publishing institutions, publishing journals, authors, keywords, and burst keywords and co-citation analysis. Our work provides a comprehensive list of landmark publications in TRPV and cardiovascular disease-related research and recognizes the contributions of critical authors and institutions. In addition, we summarize the trends and research directions in TRPV and cardiovascular disease-related research. Currently, calcium channels, metabolism, inflammation, myocardial remodeling, endothelial cell dysfunction, and the TRPV channel family remain the main research directions in this field. Studies on TRPV channel expression upregulation and dysfunction will be a hot topic in the future. Given the increasing burden of cardiovascular disease worldwide and the depth of human research on the TRPV channel, the interest in TRPV channels will continue to grow. Research on the TRPV channel will ultimately change how more clinical treatments are administered.

Regarding collaboration between authors, it shows that most collaboration occurs within continents, while cooperation between continents is rare. The same phenomenon is revealed when analyzing joint authors of organizations, where clusters offer intricate links within countries and fewer links between clusters. These results suggest that cooperation should be strengthened between continents.

## Data availability statement

The original contributions presented in the study are included in the article/Supplementary material, further inquiries can be directed to the corresponding authors.

## Author contributions

LZ: conceptualization, methodology, data curation, investigation, formal analysis, and writing – original draft. YX: data curation, software, and writing – original draft. YM: data curation and visualization. TX: visualization. CL: supervision, validation, and writing – review and editing. QL: conceptualization, funding acquisition, resources, supervision, and writing – review and editing. All authors contributed to the article and approved the submitted version

## Funding

This study was supported by the Hunan Province Innovative Project (no. 2020SK1013), the National Natural Science Foundation of China (no. 82070356), and the Natural Science Foundation Hunan Province China (nos. 2021JJ30033 and 2021JJ40870).

## Conflict of interest

The authors declare that the research was conducted in the absence of any commercial or financial relationships that could be construed as a potential conflict of interest.

## Publisher’s note

All claims expressed in this article are solely those of the authors and do not necessarily represent those of their affiliated organizations, or those of the publisher, the editors and the reviewers. Any product that may be evaluated in this article, or claim that may be made by its manufacturer, is not guaranteed or endorsed by the publisher.

## Abbreviations

TRPV: Transient receptor potential cation channel, subfamily V
TRPA: Transient receptor potential cation channel, subfamily A
TRPC: Transient receptor potential cation channel, subfamily C
NIEHS: National Institute of Environmental Health Sciences
CGRP: Calcitonin gene-related peptide
HNO: Nitroxyl
NOS: Nitric oxide synthase
TRPA1: Transient receptor potential cation channel, subfamily A, member 1
DOI: Digital Object Identifier
IF: Impact factor
CVD: Cardiovascular disease
